# Clinical Profiles and Outcomes of Adult Patients With Central Nervous System Infections: A Descriptive Cross-Sectional Study at a Single Private Tertiary Hospital in Quezon City, Philippines

**DOI:** 10.7759/cureus.103560

**Published:** 2026-02-13

**Authors:** Lee Matthew L Ponce, Minette Claire O Rosario

**Affiliations:** 1 Infectious Diseases, St. Luke's Medical Center, Quezon City, PHL

**Keywords:** brain abscess, central nervous system (cns) infections, encephalitis, epidural abscess, meningitis, subdural empyema, ventriculoperitoneal shunt infection

## Abstract

Central nervous system infections (CNSI) remain a significant cause of morbidity and mortality worldwide, yet data on local epidemiology, causative agents, and antimicrobial susceptibility patterns are limited. This paper presents a census of observed CNSI cases at a single institution to inform future studies.

To address these objectives, we conducted a retrospective cross-sectional study of adult patients with CNSI admitted from January 2017 to December 2024. We included all spectrums of CNSIs. We accessed patient charts using the hospital's electronic databases. We gathered data on demographics, clinical manifestations, risk factors, diagnostic methods, type and likelihood of CNSI, cerebrospinal fluid (CSF) analysis and measurements, etiologic agent, antimicrobial susceptibilities, treatment given, and outcomes.

Out of 357 charts reviewed, 184 met the inclusion criteria, with fever (64.7%), headache (43.5%), and neurologic deficits (36.4%) as the most common manifestations. The majority had elevated opening pressures (≥20 cm H₂O), CSF lymphocytic pleocytosis, elevated protein (≥50 mg/dL), and hypoglycorrhachia (CSF/serum glucose ratio ≤0.5). Microbiologic CSF analysis had a low positivity rate of 25%. A mixture of bacterial, fungal, viral, and parasitic agents was identified.

Tuberculous (TB) meningitis remains the most common CNSI observed. Not all patients displayed CSF pleocytosis or hypoglycorrhachia, and possible explanations include an ineffective mounting of a proper immune response in the immunocompromised or early CSF collection before an inflammatory response has occurred. Factors identified as leading to a low CSF culture positivity rate include early antibiotic initiation and fastidious organisms as causative agents. Delayed lumbar puncture or drainage is inevitable in some cases; hence, prompt antibiotic therapy is important to reduce morbidity and mortality.

## Introduction

Central nervous system infections (CNSIs) remain a significant cause of morbidity and mortality worldwide. In Southeast Asia, roughly 10.5 million people are afflicted annually [1]. In the Philippines, tuberculous (TB) and bacterial meningitis remain the top two causes of CNSI [2]. Certain factors increase the likelihood of developing CNSI, including extremes of age, comorbid conditions, malignancy, organ transplantation, human immunodeficiency virus (HIV) infection, and immunosuppressants [3]. Despite this, data regarding CNSI is lacking both locally and globally. The latest guidelines published by the Infectious Diseases Society of America (IDSA) on bacterial meningitis were in 2004. The recommendations back then may no longer be accurate due to ongoing evolution in microbes concerning antimicrobial resistance. In addition, these guidelines are based on data from foreign patients. Little data is available on local epidemiology, common causative agents, and their antimicrobial susceptibility patterns. In the context of increasing antimicrobial resistance, there is a need to reevaluate current guidelines for managing CNSIs. However, this will be difficult if data on incidence, causative agents, and their corresponding antibiotic susceptibility patterns are lacking. Furthermore, studies on CNSIs in the country are scarce [4]. Jalipa et al. reported that there are only 83 publications in the country in the past 30 years. There were also very few reports on the incidence of CNSI and its associated etiologic agents, and these reports only covered the pediatric population [5]. A study by Punsalan et al. compiled data from nine neurology training institutions in the Philippines to determine the types of CNSI and reported that TB meningitis, followed by bacterial meningitis, remains the top disease affecting the central nervous system (CNS) in the country. However, this study used data from 2011 to 2012 [2]. This paper aims to provide a census of observed CNSI cases at a single institution and provide data regarding this disease entity for future studies.

This research was previously presented as a poster abstract at the 47^th^ Philippine Society for Microbiology and Infectious Diseases (PSMID) Annual Convention held on November 18-20, 2025, in Manila, Philippines.

## Materials and methods

Study design

This research is a retrospective descriptive quantitative cross-sectional study of participants aged 18 years and above who were admitted and managed as cases of CNSI from January 2017 to December 2024. We chose this study design since our main goal was to provide a census from previously existing data of CNSI patients. The study included all spectrums of CNS infections, including meningitis, encephalitis, focal neurologic infections (brain abscess), subdural empyema, epidural abscess, suppurative intracranial thrombophlebitis, and infections of cerebrospinal fluid (CSF) and ventriculoperitoneal (VP) shunt drains. 

Criteria for subject selection

To be eligible, a patient must be at least 18 years old, have been admitted to St. Luke's Medical Center, Quezon City, Philippines, between January 2017 and December 2024, and have been diagnosed and managed for CNSI during admission. Patients managed as CNSI for more than one admission period and discharged recovered/improved were counted as separate entries, but in computing the outcomes (mortality, discharge), only the most recent one was counted. In addition, participants should have undergone CSF analysis or have submitted intraoperative cultures during their hospital stay. It was required that these results be accessible for follow-up (data should be available on electronic databases at any time during the conduct of the study). The only exception was suppurative intracranial thrombophlebitis, which relied mainly on imaging for diagnosis. Because clinical outcomes were difficult to determine, we excluded patients managed as CNSI but transferred to another institution before submitting CSF or intraoperative cultures and/or treatment. We also excluded patients whose records were incomplete or inaccessible, since the study relied on chart review.

Operational definitions

Bacterial Meningitis (Suspected)

A case with sudden onset of fever (> 38.5 °C rectal or > 38 °C axillary) accompanied by one of the following signs: neck stiffness, altered consciousness, or other meningeal signs as defined by the WHO [6].

Bacterial Meningitis (Probable)

A suspected bacterial meningitis case showing turbid, cloudy, or purulent CSF; CSF leukocyte count over 10 cells/mm^3^; or bacteria identified by Gram stain in CSF, as defined by WHO [6].

Bacterial Meningitis (Confirmed)

A suspected or probable bacterial meningitis case confirmed by culturing or identifying bacteria (i.e., polymerase chain reaction (PCR), immunochromatographic dipstick, or latex agglutination) in the CSF or blood as defined by WHO [6].

Viral Meningitis (Suspected)

A case with fever(> 38.5 °C) and one or more of the following: neck stiffness, severe unexplained headache, neck pain plus two or more of the following (photophobia, nausea, vomiting, abdominal pain, or pharyngitis with exudates)[7].

Viral Meningitis (Probable)

A suspected viral meningitis case with one or more of the following: Normal CSF glucose and normal or mildly increased CSF protein (>50mg/dL), or a moderate CSF cell increase (<500/mm^3^) with lymphocyte predominance (>50%), and an epidemiological link to a confirmed case [7]. 

Viral Meningitis (Confirmed)

A suspected or probable viral meningitis case with laboratory confirmation (CSF positive for viral genomic sequences using PCR or culture) [7].

TB Meningitis (Suspected)

Patients with clinical manifestations of meningitis, including fever, headache, vomiting, neck stiffness, irritability, convulsions, focal neurological deficits, altered consciousness, or lethargy, plus a Lancet Consensus Score ranging from 6 to 9 without imaging, or 6-11 with imaging [8].

TB Meningitis (Possible)

Patients meeting suspected TB meningitis case criteria PLUS a Lancet Consensus Score of >10 without imaging (computed tomography (CT) scan or magnetic resonance imaging (MRI)) or >12 with imaging, with at least 2 points from imaging or CSF consensus score [8].

TB Meningitis (Confirmed)

Patients who met the clinical entry criteria of suspected/possible TB meningitis PLUS either of the following: 1) CSF positive for acid-fast bacilli (AFB), MTB PCR, or culture of *Mycobacterium tuberculosis *or 2) observation of AFB in conjunction with histological changes indicative of TB in the spinal cord or brain, along with signs and symptoms, visible meningitis, and CSF changes (noted during autopsy) [8].

Neurosyphilis (Probable)

Patients meeting all of the following criteria: 1) clinical signs consistent with any of the neurosyphilitic syndromes (syphilitic meningitis, meningovascular, parenchymatous, or ocular syphilis), 2) a reactive serum treponemal test, and 3) elevated CSF protein or leukocyte count without other known causes [9].

Syphilitic Meningitis

Patients with reactive serum tests for syphilis with subacute onset symptoms falling into either of three variants: 1) acute hydrocephalus with signs not attributable to increased intracranial pressure (ICP), 2) meningitis of the vertex manifesting with seizures, decreased sensorium or focal neurologic deficits, and 3) basilar meningitis with cranial nerve palsies (III, VI, VII, and VII) [9].

Meningovascular Syphilis

Neurosyphilis syndrome, which primarily manifests with symptoms related to infarction (commonly with contralateral hemiplegia or hemiparesis, aphasia, or homonymous hemianopsia) [9].

Parenchymatous Syphilis

Neurosyphilis mainly presenting as either general paresis (insidious onset neuropsychiatric disturbances with cognitive function decline and loss of motor function) or tabes dorsalis (symptoms resulting from demyelination of the posterior spinal cord, like ataxic wide-based gait, paresthesias, urinary and fecal incontinence, loss of proprioception and vibration, and diminished knee and ankle reflexes) [9].

Ocular Syphilis

Uveitis, which may be a part of neurosyphilis and manifest as episcleritis, vitritis, retinitis, acute retinal necrosis, or retinal detachment [9].

Neurosyphilis (Confirmed)

Patients with clinical signs consistent with neurosyphilis and a reactive serum treponemal test plus either of the following: 1) a reactive Venereal Disease Research Laboratory (VDRL) in CSF, 2) detection of *Treponema pallidum* DNA in CSF or tissues with PCR, or 3) identification of treponemes in tissue with silver or immunohistochemical staining [9].

Encephalitis (Possible)

A patient with altered mental status (decreased or altered consciousness, lethargy, or personality change lasting > 24 hours with no alternative cause identified) plus at least 2 of the following: 1) fever (> 38 °C) within 72 hours before or after presentation, 2) generalized or partial seizures not entirely attributable to a preexisting seizure disorder, 3) new onset of focal neurologic findings, 4) CSF WBC >5 cells/mm^3^, 5) abnormality of brain parenchyma on neuroimaging suggestive of encephalitis that is either new or of acute onset, or 6) abnormality in electroencephalography consistent with encephalitis and is not attributable to another cause [10].

Encephalitis (Confirmed)

A possible encephalitis case with one of the following: 1) pathologic confirmation of brain inflammation consistent with encephalitis, 2) defined pathologic, microbiologic, or serologic evidence of acute infection with a microorganism strongly associated with encephalitis from an appropriate clinical specimen, or 3) absence of laboratory evidence of an autoimmune condition strongly associated with encephalitis [10].

Cryptococcal Meningitis

Patients with signs of CNSI whose CSF tested positive for India ink examination (budding encapsulated yeasts), *Cryptococcus neoformans* culture, or cryptococcal antigen lateral flow antigen test [11].

Cerebral Toxoplasmosis (Presumptive)

Patients with HIV infection and signs of CNSI with one or more cerebral mass lesions on cranial imaging not attributable to alternative diagnoses or positive serum IgG antibody to Toxoplasma [12].

Cerebral Toxoplasmosis (Definite)

A case of presumptive cerebral toxoplasmosis with either evidence of *Toxoplasma gondii* in brain biopsy or *Toxoplasma* ​​​​​​*gondii *DNA in the CSF by PCR [12].

Brain Abscess

A patient with signs of CNSI with cranial imaging findings of focal intracerebral infection beginning as a localized area of cerebritis and developing into a collection of pus surrounded by a well-vascularized capsule [9].

Subdural Empyema

Any patient with signs of CNSI whose imaging findings show a collection of pus in the space between the dura mater and the arachnoid [9].

Epidural Abscess

Any patient with signs of CNSI with imaging findings showing a localized collection of pus between the dura mater and the overlying skull or vertebral column [9].

Suppurative Intracranial Thrombophlebitis

Patient with signs of CNSI and imaging findings confirming the presence of an endovascular thrombus in the dural sinuses in the setting of a bacterial or fungal infection [9].

Cerebrospinal Drain

Temporary catheters that divert CSF externally; the proximal end is in the cerebral ventricle, subdural space, intracranial cyst, or the lumbar subarachnoid space. The distal end is connected to a collecting system and a collection bag [13].

VP Shunt

Permanent catheters with the proximal end in the cerebral ventricle, subdural space, intracranial cyst, or lumbar subarachnoid space. The distal end terminates in the peritoneal space [13].

CSF/VP Shunt Infection (Contamination)

Patients with CSF/VP shunt and signs of CNSI whose CSF aspirated from the shunt showed a positive Gram stain or culture but had normal cell counts, CSF glucose, and protein [9].

CSF/VP Shunt Infection (Suspected)

Patients with CSF/VP shunt and signs of CNSI whose CSF showed progressively increasing pleocytosis or hypoglycorrhachia on serial taps without positive CSF gram stain or cultures [9].

CSF/VP Shunt Infection (Definite)

Patients with CSF/VP shunt and signs of CNSI whose CSF aspirated from the shunt showed positive CSF culture for a pathogenic organism accompanied by significant hypoglycorrhachia or pleocytosis [9]. 

Immunocompromised State

Patients with known HIV, malignancy (ongoing therapy or past therapy); underwent splenectomy or hashyposplenism, transplant recipients, or are actively on immunosuppressants or steroids.

Meningoencephalitis (ME) Panel

An FDA-cleared syndromic test that targets 14 of the most common bacterial, viral, and fungal agents of CNS infections [14].

Outcomes

Expired patients who died due to active CNSI, another infection outside the CNS, or a non-infectious cause; Discharged patients sent home who completed treatment/opted to finish treatment outside the hospital; Transferred patients transferred for any reason after submission of CSF or intraoperative cultures, and after receiving empiric treatment (antibiotics or surgical).

Study procedure

We identified patients aged 18 and above, admitted from January 2017 to December 2024, who were managed as CNSI cases and underwent lumbar puncture. We used purposive sampling to reach the recommended number of participants, which was considered adequate as the study is descriptive quantitative in nature and does not seek generalizable claims. Blinding was not employed for the same reason. We accessed potential recruits' charts using the hospital's electronic databases, MD-Portal (MD-Portal (Pty) Ltd, Cape Town, South Africa) and DigiChart (DigiChart (Pty) Ltd, Pretoria, South Africa). We collected general data, clinical manifestations, risk factors, diagnostic methods, CNSI type, and likelihood (probable, possible, definite), CSF findings, etiologic agents, antimicrobial susceptibilities (for bacterial and fungal isolates), treatments, and outcomes. For cases with multiple positive results in the ME panel, the organisms were tallied separately but counted only as one admission. With regards to imaging findings, we reviewed cranial scans and official results noted findings suggestive of CNSI, such as meningeal enhancement, fluid collections, and increased signal intensities on the brain lobes as described by Benett et al [9]. 

Sample size 

We calculated the sample size based on the estimated population proportion with CNS infection and TB meningitis as the etiology, yielding the largest sample size among all outcomes. Assuming the proportion of TB meningitis among patients with CNSI is 43% [2], with a maximum allowable error of 6% and 90% reliability, the minimum required sample size was 184 participants.

Statistical analysis methods

We encoded data in Microsoft Excel (Microsoft Corp., Redmond, WA, USA) and analyzed them using IBM SPSS Statistics version 27 (IBM Corp., Armonk, NY, USA). Since this is a descriptive quantitative cross-sectional study, the collected data were analyzed using frequencies and percentages for categorical data and means and standard deviations for continuous data. We used a 95% confidence interval (CI) for the percentage and calculated the mean.

Ethical considerations

All study-related documents were kept and stored by the Principal Investigator in strict confidentiality for at least five years; after which they will be shredded. The principal investigator was the main person responsible for data storage. Electronic data related to the study was stored on the primary investigators' laptops, password-protected and accessible only to members of the study team. 

The study abided by the Principles of the Declaration of Helsinki (2013) and was conducted in accordance with the Guidelines of the International Conference on Harmonization-Good Clinical Practice (ICH-GCP). The Clinical Protocol and all relevant documents were reviewed and approved by the SLMC Institutional Ethics Review Committee. Patient confidentiality was respected by ensuring the anonymity of patient records.

## Results

**Figure 1:**
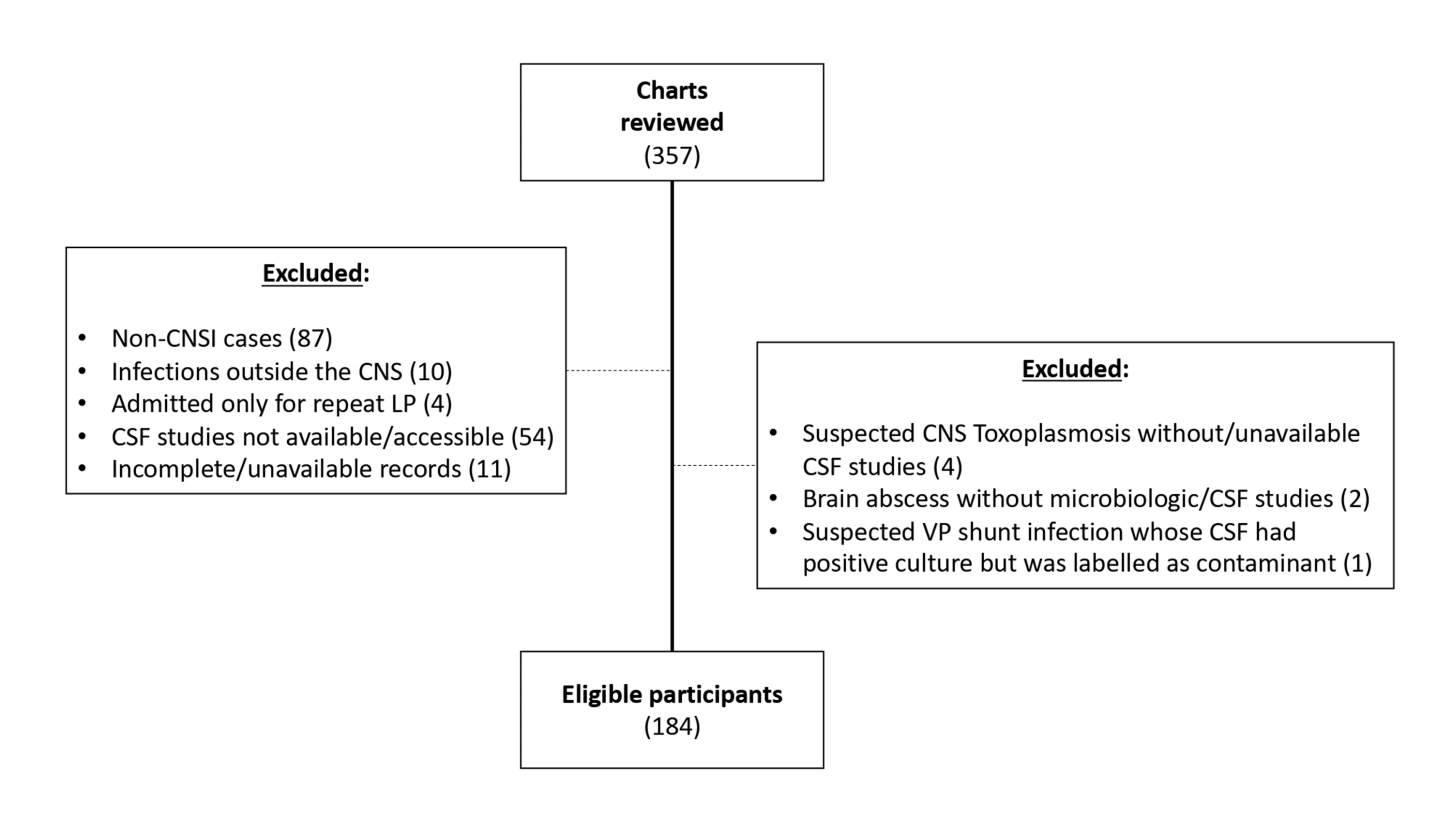
Flowchart showing recruitment of participants in the study CNSI: central nervous system infection; CNS: central nervous system; LP: lumbar puncture; VP: ventriculoperitoneal

Figure 1 summarizes the flow of participant recruitment in this study. We reviewed a total of 357 charts. Of these, 173 did not meet the inclusion criteria. Eighty-seven charts were cases without CNSI. Ten charts were due to conditions not affecting the CNS (e.g., psoas/paraspinal abscess without epidural extension, osteomyelitis of skull/vertebrae, infected subgaleal hematoma). Four cases were already under ongoing treatment for CNSI and were admitted for repeat lumbar tap. CSF studies were not performed or were unavailable for 54 cases, whereas 11 charts were incomplete or could not be accessed and were, therefore, excluded. 

Seven cases were managed as CNSI but did not meet the inclusion criteria. Four cases of possible CNS toxoplasmosis were diagnosed on clinical grounds and imaging, but no CSF analysis was done, or was not available. Two cases of brain abscess were not drained, and no CSF studies were done. A patient with a VP shunt whose CSF culture grew *Staphylococcus capitis* was also deemed a contaminant and not treated. We excluded this patient from the list. 

The number of eligible cases amounted to 184. The mean age was 47 years; the youngest recruit was 18, whereas the oldest was 94. The majority were male (67.4%). In terms of area of residence, 70.1% resided within Metro Manila. Around 27.7% lived in the northern provinces of the Philippines. One participant lived in the southern part of the Philippines, and three were from other countries, namely, the Marshall and the Solomon Islands.

Clinical manifestations

Table 1 details the most common clinical manifestations encountered in the study. Fever was the most common sign (64.7%) across all CNSI types. Over a third of the total participants presented with neurologic deficits. This list included slurred speech, diplopia, facial palsy, numbness, and extremity weakness. The neurologic deficit was the initial presentation of patients with spinal epidural abscesses. A decrease in sensorium ranked fourth among manifestations and was most commonly observed in patients with encephalitis and subdural empyema. Other signs and symptoms noted were vomiting, seizures, and nuchal rigidity.

**Table 1:** Presenting signs and symptoms of adult patients with CNSI admitted to St. Luke’s Medical Center, Quezon City, from January 2017 to December 2024. CNSI: central nervous system infection; SIT: suppurative intracranial thrombophlebitis

Clinical Manifestation	Meningitis (n=113)	Encephalitis (n=38)	Shunt Infections (n=9)	Brain Abscess (n=3)	Epidural Abscess (n=16)	Subdural Empyema (n=4)	SIT (n=1)	TOTAL (n=184)
									Fever	74	22	8	2	8	4	1	119 (64.7%)
Headache	56	16	1	3	0	4	0	80 (43.5%)
Neurologic Deficits	39	12	2	1	8	4	1	67 (36.4%)
Decreased Sensorium	39	20	4	0	0	3	0	66 (35.9%)
Vomiting	29	11	1	1	0	2	0	44 (23.9%)
Seizures	14	17	3	2	0	1	0	37 (20.1%)
Nuchal Rigidity	28	7	0	1	0	0	0	36 (19.6%)
Skin Lesions	4	8	0	0	0	0	0	12 (6.5%)

Twelve patients presented with skin lesions that may relate to the causative agent. For instance, five patients found to have encephalitis due to varicella zoster presented with vesicular lesions in the face or trunk. One patient who had maculopapular rashes on the trunk was worked up for neurosyphilis.

Comorbid conditions

Table 2 summarizes observed comorbidities. Four patients had a history of head trauma or were victims of vehicular accidents and subsequently developed post-traumatic meningitis or had shunts implanted, which became infected. Seventeen patients had recent CNS surgery. Most of them had intracranial hemorrhage or ruptured aneurysms requiring craniotomy or aneurysm coiling. Patients who had shunts inserted due to increased ICP from brain tumors were also included here.

**Table 2:** Comorbidities of all adult patients with CNSI admitted to St. Luke’s Medical Center, Quezon City, from January 2017 to December 2024. a: Head trauma, vehicular accident; b: IgE immunodeficiency, alpha thalassemia, polycythemia vera, liver cirrhosis, pregnancy; c: Patients whom HIV screening/confirmatory tests were not done but found to have low CD4 counts; d: EPTB sites including lymph node, psoas abscess, TB arteritis, Potts disease and gastrointestinal TB; e: Otitis media, sinusitis, mastoiditis. CNS: central nervous system; TB: tuberculosis; PTB: pulmonary tuberculosis; ETPB: extrapulmonary tuberculosis

Comorbidities (n=184)	Frequency (%)
Trauma^a^	4 (2.2%)
Previous CNS Surgery	17 (9.2%)
Immunocompromised State	
Malignancy	9 (4.9%)
HIV	12 (6.5%)
On Steroids/Immunosuppressants	4 (2.2%)
Others^b^	9 (4.9%)
Low CD4 Count^c^	12 (6.5%)
Recent Travel	10 (5.4%)
Ongoing TB Treatment (PTB & EPTB^d^)	30 (16.3%)
Active Head and Neck Infection^e^	8 (4.3%)
Hypertension	63 (34.2%)
Type 2 Diabetes Mellitus	36 (19.6%)
Chronic Kidney Disease	5 (2.7%)
Bronchial Asthma	10 (5.4%)

Several patients had immunocompromising conditions. Nine had active malignancy (hepatocellular, breast, lymphoma, colon, nasopharyngeal, or ovarian cancer). Twelve were HIV positive (known cases of HIV or diagnosed during admission) and managed for CNS toxoplasmosis, as well as TB and cryptococcal meningitis. An equal number of patients suspected of opportunistic CNSIs (TB or cryptococcal meningitis) could not be screened for HIV, either due to refusal or being too ill for counseling. Immunodeficiency panels, instead, revealed low CD4 counts, so they were similarly classified. Other immunocompromising conditions included chronic steroid use (for lupus nephritis, neuromyelitis optica, or post kidney transplant), pregnancy, liver cirrhosis, and blood dyscrasias (polycythemia vera, alpha-thalassemia).

Other infections present upon hospitalization were also identified. Thirty patients had tuberculosis (pulmonary and extrapulmonary). Among patients with meningitis, brain abscess, subdural empyema, and suppurative intracranial thrombophlebitis, eight had an active/recent head and neck infection (otitis, mastoiditis, sinusitis).

Ten participants had recent travel (<3 months) at the time of presentation. Medical records did not expound on travel activities. Among other comorbidities, 34% had hypertension, 19.6% had diabetes, and a few had bronchial asthma or chronic kidney disease.

CSF analysis

Tables 3-4 summarize CSF analysis variables in patients with meningitis and encephalitis, respectively. Twenty-nine patients did not undergo lumbar puncture; this group included those with shunt infections (where CSF was aspirated only from the ventricular drain), brain abscess, epidural abscess, subdural empyema, and suppurative intracranial thrombophlebitis.

**Table 3:** Summary of CSF analysis of all adult patients with meningitis admitted at St. Luke’s Medical Center, Quezon City, from January 2017 to December 2024. a: One case has viscous CSF, hence opening pressure was not measured; n/a: not applicable since cell type is a categorical variable; SD: standard deviation CSF: cerebrospinal fluid

CSF Analysis Parameters		Bacterial Meningitis^a^ (n=37)			TB Meningitis (n=49)			Fungal Meningitis (n=22)			Parasitic Meningitis (n=5)		
		Frequency	Mean ± SD	Median (Range)	Frequency	Mean ± SD	Median (Range)	Frequency	Mean ± SD	Median (Range)	Frequency	Mean ± SD	Median (Range)
Opening Pressure (cmH_2_0)	<20	17	21.54 ± 10.02	20 (2.5 to 45)	15	24.29 ± 10.91	22.25 (5 to 55)	9	28.29 ± 15.27	24.75 (5 to 55)	5	14 ± 4.36	15 (9 to 19)
	≥20	19			33			13			0		
WBC Count (cells/mm^3^)	Acellular	5	14,984.56 ± 82,652.02	24 (0 to 496,360)	3	117.92 ± 154.20	56 (0 to 800)	6	92.09 ± 234.36	22 (0 to 1104)	2	24 ± 31.50	8 (0 to 72)
	1-499	24			44			15			3		
	500-999	1			0			0			0		
	≥1000	7			2			1			0		
Predominant Cell Type	Neutrophils	10	n/a	n/a	0	n/a	n/a	0	n/a	n/a	0	n/a	n/a
	Lymphocytes	22			46			16			3		
	None	5			3			6			2		
CSF Protein (mg/dL)	<50	10	228.94 ± 283.94	78 (26 to 1,097)	4	181.63 ± 146.86	142 (36 to 722)	3	94.68 ± 56.02	77.50 (31 to 221)	0	87.20 ± 49.91	69 (57 to 176)
	50-199	14			27			17			5		
	≥200	13			18			2			0		
CSF/Serum Glucose Ratio	<0.5	22	0.40 ± 0.23	0.42 (0.01 to 0.70)	29	0.44 ± 0.19	0.47 (0.05 to 0.88)	14	0.36 ± 0.19	0.40 (0.01 to 0.72)	1	0.55 ± 0.09	0.52 (0.47 to 0.69)
	≥0.5	15			20			8			4		

**Table 4:** Summary of CSF analysis of all adult patients with viral encephalitis admitted at St. Luke’s Medical Center, Quezon City, from January 2017 to December 2024. b: turned positive for both bacterial and viral pathogens in the meningoencephalitis panel; n/a: not applicable since cell type is a categorical variable; SD: standard deviation CSF: cerebrospinal fluid

CSF Analysis Parameters		Viral Encephalitis (n=38)		
		Frequency	Mean ± SD	Median (Range)
Opening Pressure (cmH_2_0)	<20	21	19.45 ± 7.89	19 (7 to 40)
	≥20	17		
WBC Count (cells/mm^3^)	Acellular	7	118 ± 296.2	28 (0 to 1720)
	1-499	29		
	500-999	1^b^		
	≥1000	1		
Predominant Cell Type	Neutrophils	1^b^	n/a	n/a
	Lymphocytes	30		
	None	7		
CSF Protein (mg/dL)	<50	11	109.30 ± 133.22	62 (28 to 720)
	50-199	24		
	≥200	3		
CSF/Serum Glucose Ratio	<0.5	8	0.60 ± 0.14	0.62 (0.21 to 0.87)
	≥0.5	30		

Most cases with elevated opening pressures were seen in bacterial, TB, and fungal meningitis, reaching as high as 55 cmH_2_0. There was one case of meningitis in which the opening pressure was not measured due to high CSF viscosity; the culture eventually grew positive.

Most samples had WBC counts between 1 and 499 cells/mm^3^. Acellular taps accounted for 14% of meningitis cases and 18% of encephalitis cases. The rest had counts above 500 cells/mm^3^, and nearly all were positive in either culture or CSF PCR.

One case had positive results on a bacterial and viral ME panel and elevated CSF WBC levels. In CSF, lymphocytes were the predominant cell type observed. Only 7.2% of the combined meningitis and encephalitis CSF samples showed neutrophil predominance, and almost all of these cultures grew bacteria. The remaining 14% were acellular taps; hence, no predominant cell type was seen.

The CSF protein level averaged 158.83 mg/dL (range: 26-1097 mg/dL) for meningitis cases and 109.30 mg/dL (range: 28-720 mg/dL) for encephalitis cases. Most participants (57%) had levels in the 50-199 mg/dL range. Those with levels above 200 mg/dL (23.8%) had bacterial or TB meningitis, as shown by positive CSF cultures. The remaining had CSF protein below 50 mg/dL.

We recorded the CSF/serum glucose ratio to avoid falsely low or high CSF glucose levels due to hypo- or hyperglycemia. Using a 0.5 cutoff, low glucose was most common in bacterial, TB, and fungal meningitis, with these cases testing positive by culture or CSF PCR.

Diagnostic test positivity

Table 5 shows the proportion of patients with positive CSF microbiologic results across the various CSF microbiologic studies and imaging modalities. Only 21 patients had positive microscopy (Gram stain, acid-fast stain, India ink) for microorganisms. Forty-seven (25.5%) had positive CSF/abscess aspirate cultures.

**Table 5:** Diagnostic test positivity rates for CSF analysis and imaging among all adult patients with CNSI admitted at St. Luke’s Medical Center, Quezon City, from January 2017 to December 2024. a: Includes gram stain, acid-fast stain, and India ink; b: Not included in meningoencephalitis panel CSF: cerebrospinal fluid; CNSI: central nervous system infection; MTB PCR: *Mycobacterium tuberculosis* polymerase chain reaction; CALAS: Cryptococcal Antigen Latex Agglutination System; FTA-ABS: fluorescent treponemal antibody absorption

Diagnostic Test (n=184)	Positive (%)
Microscopy^a^	21 (11.4%)
Culture	47 (25.5%)
Polymerase Chain Reaction	
Meningoencephalitis Panel	31 (16.8%)
Japanese Encephalitis PCR^b^	1 (0.5%)
MTB PCR	10 (5.4%)
Antigen/Antibodies	
Bacterial Antigen (Phadebact)	3 (1.6%)
CALAS	26 (14.1%)
FTA-ABS	1 (0.5%)
Toxoplasma IgG	2 (1.1%)
Cranial/Spinal Imaging	87 (47.5%)

Building on the culture data, additional diagnostic methods provided further insights. In addition to cultures, 42 patients had positive CSF PCR results. Of these, 31 (16.8%) tested positive on the ME panel. Most were viral encephalitis, followed by bacterial meningitis. One case was positive for both a viral and bacterial agent. Another tested positive for CSF Japanese encephalitis virus PCR, which was not in the ME panel. Ten cases were positive for *Mycobacterium tuberculosis* PCR (CSF and epidural abscess), with half also yielding culture-positive results.

Transitioning to other diagnostic approaches, evaluation for CSF pathogen antigens revealed three cases positive for bacterial antigens, 26 for cryptococcal antigen latex agglutination system (CALAS), one for fluorescent treponemal antibody absorption (FTA-ABS), and two for Toxoplasma IgG. Nearly half of the patients (47.3%) exhibited imaging findings suggestive of infection, including leptomeningeal enhancement, cystic brain lesions, or fluid collections consistent with abscess.

Likelihood of CNS Infection

Table 6 lists the breakdown of CNS infections by likelihood. For bacterial meningitis, there were 14 confirmed cases (all with positive cultures or PCR), eight possible, and 15 probable. Patients considered as cases of neurosyphilis suspects were included in the list of probable cases due to a lack of access to CSF VDRL. We classified CSF samples that tested positive for *Mycobacterium tuberculosis* PCR or yielded positive cultures as confirmed cases of TB meningitis. We subjected those who did not meet these criteria to the Lancet Consensus Scoring System. We labeled four cases that scored at least 12 as possible TB meningitis, while 30 scored less than 11 and were labeled as suspected TB meningitis. Of the fungal meningitis cases, 14 had either positive cultures or positive ME panels, which were considered confirmed cases. The remaining 8 cases were based solely on CALAS titers and were therefore tagged as suspected fungal meningitis. Finally, the parasitic meningitis (all CNS toxoplasmosis cases) was divided into two groups based on CSF toxoplasma IgG positivity. Only 2 cases had positive CSF antibodies, so they were labeled as confirmed cases, and the rest were labeled as suspected cases.

**Table 6:** Tally of CNS infections admitted at St. Luke’s Medical Center, Quezon City, from January 2017 to December 2024. a: Included cases of neurosyphilis where CSF VDRL is unavailable/cannot be accessed; b: Classification as possible or probable based on the Lancet Consensus Scoring System; c: Cannot be classified due to lack of CSF analysis (WBC count, glucose, and protein); d: Based on imaging alone, CSF analysis not done. CSF: cerebrospinal fluid; CNS: central nervous system; VDRL: Venereal Disease Research Laboratory; VP: ventriculoperitoneal

CNS Infection	Etiologic Agent	No	Probable/ Suspected	Possible	Definite/ Confirmed
						Meningitis (n=113)	Bacterial	37	15^a^	8	14
Tuberculous^b^	49	30	4	15		
Fungal	22	8	0	14		
Parasitic	5	3	0	2		
Encephalitis (n=38)	Viral	38	0	20	18
CSF/VP Shunt Infection (n=9)	Bacterial	9^c^	0	0	0
						Brain Abscess (n=3)	Bacterial	2	0	0	2
Fungal	1	1	0	0							
Epidural Abscess (n=16)	Bacterial	13	0	0	13	
	Tuberculous	3	0	1	2
Subdural Empyema (n=4)	Bacterial	3	0	0	3
	Tuberculous	1	0	0	1
Suppurative Intracranial Thrombophlebitis (n=1)	Bacterial^d^	1	0	0	0

Turning to the encephalitis cases, we labeled 18 as confirmed. This group includes 15 cases with a virus detected in the ME panel and three cases with positive dengue serum antibodies, which still fall under the scope of confirmed encephalitis. Twenty cases that did not meet the criteria were classified as possible encephalitis.

Regarding CNS/VP shunt infections, these cannot be classified into the above categories since the case definitions require CSF analysis, specifically differential count, glucose, and protein. Not all patients had their CSF analyzed. Four cases had positive CSF growths, but without CSF analysis, they cannot be classified as confirmed cases.

Next, for the brain abscess cases, two underwent drainage and were confirmed intraoperatively to have purulent material, and were categorized as confirmed cases. One case was identified by imaging and clinical presentation; the patient had a lumbar tap with elevated CALAS titers and was treated as a cryptococcal abscess. A repeat MRI several weeks later showed regression, so we labeled this as a probable case.

In epidural abscess cases, all except one yielded positive cultures or were positive for MTB PCR. Hence, we classified these as confirmed cases. The sole case that did not have positive cultures or PCR was managed as a possible case of TB arachnoiditis based on imaging studies, clinical findings, and risk factors.

Addressing subdural empyema, all cases had imaging findings consistent with empyema, and all underwent source control, which confirmed the presence of purulent material in the brain. All of these were classified as confirmed cases.

Finally, for the sole case of suppurative intracranial thrombophlebitis, no lumbar puncture and CSF analysis were done. The diagnosis was mainly based on imaging findings from contrast-enhanced MRI and/or CT scans, which revealed mastoiditis with concomitant dural sinus thrombosis. There is no case definition based on likelihood; therefore, it is not classified into the above categories.

CSF microbiologic studies 

Table 7 lists the organisms identified in culture, PCR, or serology. We identified a combination of bacteria, tuberculosis, fungi, and Toxoplasma in the meningitis cases. Bacterial isolates included Gram-positive and negative organisms, some of which were common agents of meningitis (*Streptococcus pneumoniae*, *Streptococcus agalactiae*, *Neisseria meningitides*, and *Haemophilus influenzae*). There were also 15 *Mycobacterium tuberculosis* isolates (positive in culture or *Mycobacterium tuberculosis* PCR) and one non-TB mycobacterium (*Mycobacterium abscessus*). There were 11 cases of *Cryptococcus *​​​​​​*neoformans* (all CALAS-positive) among the fungal isolates, one CSF culture positive for *Candida albicans*, and two cases of culture-negative cryptococcal meningitis that were positive in the ME panel. There were also two cases of confirmed CNS toxoplasmosis whose CSF tested positive for Toxoplasma IgG. There were cases of possible neurosyphilis, but confirmatory CSF studies (CSF VDRL) were unavailable and were not included in the list.

**Table 7:** Etiologic agents of all definite/confirmed CNS infections admitted at St. Luke’s Medical Center, Quezon City, from January 2017 to December 2024, identified through culture, PCR, serology, and antigens. a: Extended spectrum betalactamase (ESBL) producing organism; b: Methicillin-sensitive isolate; c: Multi-drug resistant organism (MDRO) CNS: central nervous system; VP: ventriculoperitoneal

Meningitis	Encephalitis	CSF/VP Shunt Infections	Brain Abscess	Epidural Abscess
*Streptococcus agalactiae* (1)	Herpes Simplex Virus (3)	*Staphylococcus aureus* (1)^b^	*Proteus mirabilis* (1)	*Staphylococcus aureus* (6)^b^
*Streptococcus pneumoniae* (6)	Varicella Zoster Virus (8)	*Enterococcus faecalis* (1)		*Staphylococcus epidermidis* (1)
*Streptococcus suis* (1)	Cytomegalovirus (2)	*Enterococcus faecium* (1)		*Streptococcus agalactiae* (1)
*Enterococcus faecalis* (1)	Human Herpes Virus 6 (1)	*Serratia marcescens* (1)		*Streptococcus lugdunensis* (1)
*Bacillus cereus* (1)	Dengue Virus (4)			*Klebsiella pneumoniae* (1)
*Klebsiella pneumoniae* (2)^a^	Japanese Encephalitis Virus (1)			*Klebsiella oxytoca* (1)^c^
*Haemophilus influenzae *(2)				*Enterobacter cloacae* (1)
*Neisseria meningitides* (1)				*Proteus mirabilis* (1)
*Mycobacterium abscessus* (1)				*Pseudomonas aeruginosa* (1)
*Mycobacterium tuberculosis* (15)				*Pseudomonas putida* (1)
*Candida albicans* (1)				*Mycobacterium tuberculosis* (2)
*Cryptococcus neoformans* (11)				
*Cryptococcus *spp. (2)				
*Toxoplasma gondii* (2)				

For encephalitis, we identified six viruses. There were two cases of cytomegalovirus (CMV) encephalitis (one with elevated CSF CMV titers), three cases of herpes simplex virus (HSV) encephalitis (one is a HSV serum IgM/IgG positive kidney transplant patient, hence still labeled as confirmed encephalitis), one case of human herpes virus 6 (HHV 6), one case of Japanese encephalitis, and eight cases of varicella zoster encephalitis. Four cases of dengue encephalitis were identified (one CSF PCR positive and three with positive dengue serologies; hence, classified as confirmed encephalitis).

Shunt infections yielded only four isolates: three were Gram-positive (*Enterococcus spp*. and *Staphylococcus aureus*), and one was Gram-negative (*Serratia marcescens*). Of the three brain abscess cases, only one specimen had a positive culture, which grew *Proteus mirabilis*.

All but one epidural abscess had positive cultures (two cases even grew two microorganisms). There was a mixture of Gram-positive and Gram-negative isolates. Two cases of tuberculous epidural abscesses were positive in *Mycobacterium tuberculosis* PCR, and both had concomitant Pott's disease.

Treatment interventions and outcomes

Regarding initial empiric therapy, patients received different antimicrobial regimens. In most cases (42%), a single antimicrobial was administered (beta-lactam, isoniazid/rifampicin/pyrazinamide/ethambutol (HRZE), antiviral, antifungal, or cotrimoxazole). These include cases referred to the infectious disease service when CSF studies were available (e.g., positive CALAS or pathogen detected on the ME panel). Other cases had a focus of infection outside the CNS, suggesting the etiologic agent (e.g., vesicular rashes, hence started on acyclovir; pulmonary tuberculosis, hence started on HRZE).

The rest of the participants received a combination of antimicrobials depending on the risk factors identified, HIV positivity, patient clinical status, and initial CSF analysis. Around 38% received dual antimicrobials, 15% received triple antimicrobials, and 4% received quadruple antimicrobials. These combinations usually included beta-lactam, vancomycin, metronidazole, HZRE, antiviral agents, antifungal agents, and cotrimoxazole. Two patients did not receive antimicrobials. These were cases of dengue with positive serologies and decreased sensorium.

Upon receipt of CSF results, 44% of participants had their empiric therapies de-escalated (discontinued unneeded antimicrobials); for the remaining 56%, de-escalation was not performed. Most of these cases had negative CSF studies or had hospital courses complicated by another medical problem, making de-escalation difficult.

Source control is important in many CNS infections, so we also tallied neurosurgical interventions. The most common procedure was drainage of an abscess, performed for epidural abscesses, subdural empyemas, and brain abscesses. Among the cases of CSF shunt infection, only six underwent removal. Nine cases underwent shunt insertion to address increased intracranial pressure caused by the infection (most of which were due to TB meningitis).

Of the patients included in the study, 74.5% were discharged. Around 8.9% died (a combination of CNSI itself or another medical problem that complicated the hospital course). The remaining cases opted to transfer to another institution or be discharged against medical advice. These cases had undergone lumbar tap and were initially started on empiric antimicrobials; therefore, we still included them in the study.

## Discussion

Epidemiology of CNSIs

Data on CNSI epidemiology are limited at the local level. Punsalan et al. identified TB meningitis, bacterial meningitis, and viral encephalitis as the most common adult cases [2]. This study finds similar results: TB meningitis is most common, followed by viral encephalitis and bacterial meningitis. The high number of TB meningitis cases likely reflects the country's elevated tuberculosis incidence. The 2020 WHO Global Tuberculosis Report lists the Philippines as having the fourth-highest incidence worldwide, after India, China, and Indonesia [15]. Mycobacteria reach the CNS through the blood during transient bacteremia or when immune cells carrying acid-fast bacilli cross the blood-brain barrier (Trojan Horse mechanism) [16].

Cryptococcal meningitis ranks fourth among the infections encountered in this study and fifth in the study by Punsalan et al. [2]. In addition, over half of these cases are confirmed cases in this paper. Most cryptococcal meningitis cases in this study occurred in patients with an existing immunocompromising condition (such as a malignancy, confirmed HIV, or, if not tested, a low CD4 count). Punsalan et al. did not actively seek risk factors for cryptococcal meningitis but suggested that the rising incidence of HIV may contribute to the increase in cases [2]. Both cryptococcal and TB meningitis are considered opportunistic infections among persons living with HIV, and there are guidelines for their treatment in this population to minimize the risk of immune reconstitution [17].

Clinical manifestations

Regarding clinical manifestations, the most common signs and symptoms in this study are fever (64.7%), headache (43.5%), neurologic deficits (36.4%), decreased sensorium (35.9%), and vomiting (23.9%). These findings align with Bennet et al., who also report headache, fever, altered sensorium, and vomiting as the most frequent signs of bacterial meningitis [9,18]. Bjlsma et al. noted meningismus as a common sign in bacterial meningitis [18], but we did not observe this in our study. Meningismus is absent in CNSI that lack meningeal irritation (e.g., encephalitis, brain abscess), and meningeal signs have poor diagnostic accuracy. Thomas et al. reported that Kernig and Brudzinski signs had 5% sensitivity and nuchal rigidity only 30%, so these cannot distinguish CNSI from those without it [9,19].

One important observation in this study is the presence of skin lesions as an initial presenting symptom. Twelve patients manifested with either vesicular lesions in the body, maculopapular lesions, or prurigo nodularis. Most of those with vesicular lesions had positive CSF ME panels for the varicella-zoster virus. The rest had either low CD4 counts or tested positive for HIV, which could have predisposed them to develop CNSI. The lone case that tested positive for *Neisseria meningitides* did not have the classic purpuric rash but had an ear infection, which could explain the organism's possible source. This finding emphasizes the importance of a thorough physical exam, as the description of skin lesions may provide a clue to identifying the etiologic agent and initiating prompt therapy.

CSF analysis

Moving on to the CSF findings, 54% of patients had elevated CSF pressure (≥20 cm H_2_O), a classic finding in bacterial, cryptococcal, and TB meningitis and, in some cases, encephalitis. Those with pressures more than 30 cm H_2_0 had positive cultures or PCR results. Among those who underwent lumbar puncture, around 44% had opening pressures <20 cm H_2_0 and were primarily seen in CNS toxoplasmosis, suspected neurosyphilis cases, viral encephalitis, and some cases of bacterial and tuberculous meningitis. Regarding the WBC count of all cases who underwent lumbar tap, 76% had counts between 1 and 499 cells/mm^3^, 1.3% between 500 and 999 cells/mm^3^, and 7% greater than 1000 cells/mm^3^. All but one of the cases with a WBC of at least 500cells/mm^3^ were confirmed cases of bacterial meningitis, which is an expected finding. The remaining were acellular taps; hence, the WBC count was zero. The predominant cell type in most cases was lymphocytes (77.5%), followed by neutrophils (7%). Those with neutrophilic predominance were mainly confirmed cases of bacterial meningitis, again, an expected finding. Regarding CSF protein, most cases had elevated levels: 57% were in the 50-199 mg/dL range, and 23.8% were at least 200 mg/dL. In CSF glucose, most cases had elevated CSF to serum glucose levels, with 51% having at least 0.5. 

Inflammation from the inciting agent may explain the CSF changes in CNSIs. Once the blood-brain barrier is breached, inflammatory cells enter, causing CSF pleocytosis. The agent's interaction with these cells increases cytokine and toxin excretion, elevates CSF protein levels, and promotes cerebral edema. This edema and greater blood-brain barrier permeability raise ICP, producing headache, vomiting, and elevated opening pressures [9].

One possible explanation for patients without the expected CSF findings (CSF pleocytosis, hypoglycorrhachia) is depressed immune function. For example, Vinnard and colleagues reported that, in some studies, persons living with HIV afflicted with TB meningitis have lower CSF leukocyte counts and protein levels [20]. Similarly, some patients in their study who had HIV or low CD4 lymphocyte counts exhibited acellular taps yet had confirmed CNSI (TB and cryptococcal meningitis), illustrating this unexpected CSF finding. In another report, Hase et al. described a patient with *Neisseria* ​​​​​​*meningitides* in CSF but without CSF pleocytosis; they hypothesized that the lumbar puncture was performed at an early stage of CNSI, before an inflammatory reaction developed [21].

Regarding CSF lymphocytic pleocytosis, even in bacterial meningitis, the literature lacks clear explanations, as observed in this study (21 cases; 5 with positive cultures). In a 1985 paper, Powers noted that CSF lymphocytosis was primarily seen in neonates and patients with meningismus but occurred across all ages without any clear characteristics. He then concluded that if CSF WBC counts are below 1000 cells/mm^3^, CSF lymphocytic pleocytosis has little value in distinguishing bacterial meningitis from those caused by other organisms [22]. Despite CSF lymphocytosis, the confirmed cases of bacterial meningitis have elevated protein and low CSF glucose levels; hence, CSF studies should be evaluated as a whole rather than relying on individual components.

Despite these numbers, CNSI causes increased morbidity and mortality if treated late. Delayed antibiotics can lead to poor outcomes, especially in immunocompromised patients, where a high index of suspicion is needed. Treatment decisions for suspected CNS infections rely on clinical presentation, imaging, CSF analysis, and cultures. The Infectious Disease Society of America highlights a similar approach for ventriculitis and shunt infections, recommending correlation of CSF results with clinical signs, such as fever, seizures, or altered mentation, especially after surgery or head trauma [13].

CSF microbiologic tests

Culture or PCR is the gold standard for diagnosing CNSI from bacteria, mycobacteria, viruses, fungi, and parasites [6]. Microscopy may suffice to confirm CNSI, especially for *Mycobacterium* *tuberculosis* and *Cryptococcus *spp. [8,11]. For fastidious organisms, a positive CSF immunoglobulin (e.g., CNS toxoplasmosis, neurosyphilis) or antigen (e.g., CALAS for cryptococcal CNSI) is an alternative to culture [12].

In this study, only a small proportion of cases had positive Gram stain and culture findings. CSF Gram stain has variable sensitivity for diagnosis, ranging from 60% to 90%, and is further affected by CSF bacterial load, prior antibiotic use, and the type of organism [23]. Men et al. reported that, regardless of organism, only 25% show positive CSF microscopy results with bacterial loads <10^3^ CFU/mL, which increase to 60% with loads between 10^3^ and 10^5^ CFU/mL [24]. The same factors lower the yield of CSF cultures. The diagnosis of bacterial meningitis may even be missed in 13% of cases due to negative cultures [24]. Delays in obtaining CSF samples for culture and analysis, as well as prior antibiotic exposure, further decrease the yield. For PCR, around 47% (18 of 38) of viral encephalitis tested positive, while 10% (10 of 49) tested positive for MTB PCR. Although specific, CSF MTB PCR is not sensitive [9].

For cryptococcal meningitis, most patients who tested positive for CALAS were treated regardless of the CALAS level. CALAS has over 90% sensitivity and specificity [9]. No reliable literature-based cutoff exists for definitively diagnosing cryptococcal infection. Patients received treatment if they had a positive CALAS and either elevated opening pressure, abnormal imaging, or relevant risk factors.

Other CNSIs

For CNSI types such as shunt infection, brain abscess, epidural abscess, subdural empyema, and suppurative intracranial thrombophlebitis, most cases lacked CSF studies and were diagnosed by imaging, later confirmed with source control. Nearly all epidural abscess cases had positive cultures or PCR. Brain abscess and subdural empyema were confirmed intraoperatively by the presence of purulent material.

The challenge was shunt infections: only 44% (four of nine) had samples sent for CSF analysis (cell counts, glucose, and protein). Another 44% had positive cultures. These factors made it difficult to estimate infection likelihood using the definitions of Bennett et al.. Growth in CSF from shunt devices may be contaminants. However, the likelihood of true infection rises when repeat samples yield the same organism [9]. Also, the 2017 IDSA guidelines state that CSF abnormalities may not reliably indicate infection, and normal CSF studies do not rule it out [13].

Management of CNSIs

We observed various regimens in this study. Patients received combinations of beta-lactams, vancomycin, metronidazole, antivirals, HRZE, anti-fungals, and cotrimoxazole. Most received beta-lactam monotherapy with anaerobic coverage, or, when an ENT infection, empyema, or abscess was present, added metronidazole or clindamycin to the beta-lactam.

Antivirals were given to patients with seizures or behavioral changes, hence suspected of encephalitis. Patients with immunocompromising conditions, such as HIV, and cystic lesions on imaging suggestive of toxoplasmosis received cotrimoxazole. Empiric treatment for tuberculous meningitis was initiated when the opening pressure was elevated, and lymphocytic predominance was observed.

The causes of CNSI vary by age [9]. Apart from young or elderly patients, those with penetrating head trauma, prior neurosurgery, or who are immunocompromised, *Streptococcus *​​​​​​*pneumoniae* is most often the responsible pathogen. Therefore, a third-generation cephalosporin is recommended as a first-line agent. Vancomycin may be added if resistance is suspected [25]. If encephalitis is suspected based on clinical features or rapid CNS decline, acyclovir is initiated while awaiting CSF results [9]. For brain abscesses, antibiotics can be delayed if drainage will happen within hours and the patient is stable; otherwise, empiric treatment includes a third- or fourth-generation cephalosporin, vancomycin, and metronidazole [25]. A similar approach is used for empyema [9]. In TB meningitis, a high opening pressure (>25 cmH_2_O) is a key finding [26]. When TB is suspected or present in the body, starting HRZE is advisable due to the poor prognosis of TB meningitis [9, 26].

Much of the literature advocates de-escalation of therapy once the etiologic agent is identified. In this study, 44% (81 out of 184) de-escalated antimicrobial therapy when CSF studies and culture results were available. The remaining 56% did not de-escalate, either because no organism was identified and treatment was completed, or because other infections complicated the hospital stay.

Limitations of the study

This is a descriptive quantitive cross sectional study. As such, we make no generalizations, associations, or claims of significance.

We encountered several limitations in this retrospective study, which relied heavily on chart review. First, the data gathered depended mainly on what was available in the medical records. Data deemed significant (e.g., travel history, exposure to animals) may not be elicited or written in the chart.

Next, a retrospective chart review had limited access to necessary diagnostics. Some laboratory test specimens (e.g., CSF VDRL) must be sent to another institution and are not accessible to the researcher.

In addition, specific tests such as HIV immunoassay were not accessible due to confidentiality issues. Though a low CD4 lymphocyte count in the immunodeficiency panel may suggest an immunocompromised state, HIV status cannot be definitively confirmed.

Furthermore, the use of purposive sampling added the risk of potential selection bias. But this was not a major issue in this study since we only wanted to describe the demographic profile of patients with CNSI. Again, making generalizations, associations, or claims of significance was not part of the study objectives. 

Finally, since the electronic database mainly covers inpatient admissions, outpatient follow-ups cannot be accessed. This inaccessibility was sometimes tricky, especially when following up on a patient's management after culture results were available. Most of the data gathered covered only the hospital course.

Due to time and logistical constraints, only CNSI patient data from a single institution was tallied in this paper. This limited the sample size for analysis. Incorporating data from other institutions and doing a multi-center census of CNSI patients is encouraged for future studies. Combining information from several hospitals will increase the sample size and may provide a better description of the CNSI patient profile in the country.

## Conclusions

Among CNSIs, TB meningitis remains the most common observed in adults. Fever, headache, neurologic deficits, and decreased sensorium are the main symptoms. A number of patients with CNSI had immunocompromising conditions (malignancy, HIV, low CD4 lymphocyte count), a concurrent head and neck infection, or ongoing TB infection. A skin rash in a few participants provided clues to the causative agent and underscored the need for thorough physical exams.

Not all CSF studies among the included participants showed expected findings, such as CSF pleocytosis or hypoglycorrhachia. However, those with elevated CSF protein or low CSF glucose later had positive cultures or PCR results. Possible explanations for not meeting these findings include a weakened immune response in immunocompromised states or early CSF collection before an inflammatory response occurs.

Fewer than half of microbiologic CSF studies tested positive by microscopy and had positive cultures or PCR results. Early antibiotic use lowers yield, and fastidious organisms make recovery challenging. Sometimes pleocytosis and hypoglycorrhachia occur despite negative cultures, so consider all CSF study results collectively. While microbiologic studies matter, delays in lumbar puncture or drainage sometimes occur. Always prioritize prompt antimicrobial therapy if CNSI is suspected to reduce morbidity and mortality.
